# HOTAIR expands the population of prostatic cancer stem-like cells and causes Docetaxel resistance via activating STAT3 signaling

**DOI:** 10.18632/aging.103188

**Published:** 2020-07-13

**Authors:** Ning Wang, Yaodong Jiang, Shidong Lv, Haoran Wen, Dehua Wu, Qiang Wei, Qiang Dang

**Affiliations:** 1 Department of Radiation Oncology, Nanfang Hospital, Southern Medical University, Guangzhou 510515, Guangdong, China; 2 Department of Oncology, Dongguan Kanghua Hospital, Dongguan 523000, Guangdong, China; 3 Department of Urology, Nanfang Hospital, Southern Medical University, Guangzhou 510515, Guangdong, China

**Keywords:** HOTAIR, prostatic cancer stem-like cells, STAT3, Docetaxel resistance

## Abstract

Prostatic cancer stem-like cells (PCSLCs) play an essential role in PCa development. Accumulating evidence suggests that androgen deprivation therapy (ADT) or chemotherapy using docetaxel could expand the population of PCSLCs. Therefore, understanding the underlying mechanisms responsible for PCSLCs expansion has broadly scientific interest. Here, our results revealed that lncRNA HOTAIR could increase PCSLCs population via activating STAT3 signaling. Mechanistically, HOTAIR functioned as miR-590-5p sponge and prevented it from targeting the 3’UTR of IL-10, one upstream molecule of STAT3 signaling, leading to IL-10 upregulation and STAT3 activation. We also found that HOTAIR was required and sufficient to cause Docetaxel resistance (DocR) in C4-2 PCa cells. Moreover, our *in vivo* animal study also confirmed that Du145-HOTAIR mice had a faster tumor growth rate and a poorer survival rate compared to control cohorts. Our data build compelling rationale to target HOTAIR for the depletion of PCSLCs and alleviation of Docetaxel resistance.

## INTRODUCTION

Prostate cancer (PCa) remains the leading diagnosed cancer among men [1]. Targeting the androgen receptor (AR) signaling is the mainstay treatment owing to the significance of AR in prostate cancer development [2–4]. Nevertheless, most patients will relapse within 2 years and develop castration-resistant PCa (CRPC), a highly advanced form of PCa that is more lethal and more metastatic. The reactivation of AR in CRPC stage allows the clinical use of AR antagonists such as enzalutamide and abiraterone [5, 6]. Docetaxel (Doc) is also approved by FDA to treat metastatic CRPC [7]. Even though these treatments show promising therapeutic efficacy, drug resistance will eventually occur due to multiple mechanisms. The expansion of prostatic cancer stem-like cells (PCSLCs) is considered as one of mechanisms responsible for drug resistance [8]. However, the underlying mechanisms accounting for the expansion of PCSLCs upon drug treatments are still largely unknown.

PCa is a heterogeneous mass [9]. Cancer stem cells (CSCs) play essential role in maintaining homeostatic microenvironment of cancer [10, 11]. CSCs bear unlimited self-renewal ability and can differentiate into different types of cancer cells [10, 11]. Compared to normal cancer cells, CSCs are more malignant and easily form tumor *in vivo*. Numerous studies have already defined the role of PSCLCs in PCa progression. Previous study has demonstrated that STAT3 activation was required for PCSLCs expansion upon androgen deprivation therapy (ADT) treatment [12]. STAT3, which is activated upon the phosphorylation at Y705, can translocate to the nucleus to regulate gene expression when it complexes with cofactors [13, 14]. STAT3 signaling is tightly involved in cancer development including cancer initiation, cancer progression and drug resistance [15]. However, the driving forces for STAT3 activation in PCSLCs are still being extensively investigated.

The discovery of long non-coding RNAs (lncRNA) leads to the advance of biological research. The contributions of lncRNAs to cancer progression have been widely recognized in recent years [16]. For example, MALAT1, which was significantly increased in CRPC patients [17], bond SF2 (serine/arginine splicing factor 2) to facilitate AR-v7 production and caused Enz resistance [18]. The lncRNA HOTAIR has been shown to bind PRC2 complex to regulate gene expression [19]. HOTAIR has also been documented as a driving force for various cancer development including PCa [20–22]. The transcription of HOTAIR was suppressed by androgen receptor (AR) before castration and this suppression was released upon ADT treatment. Induction of HOTAIR could promote cell growth and cell invasion of PCa cells even in the castrated condition [20]. Mechanistically, HOTAIR interacted with AR and prevented it from MDM2 mediated protein degradation [20]. However, the role of HOTAIR in the development of PCSLCs and Doc resistance (DocR) is still elusive.

In this study, we identified that HOTAIR overexpression expanded the population of PCSLCs by activating STAT3 signaling. HOTAIR worked as miR-590-5p sponge and prevented it from binding with the 3’UTR of IL-10, leading to IL-10 upregulation. MiR-590-5p was sufficient to block HOTAIR induced STAT3 activity and HOTAIR mediated PCSLCs expansion. In addition, we also found that HOTAIR was required and sufficient to cause Doc resistance by increasing PCSLCs population. The *in vivo* study also confirmed our *in vitro* findings. Overall, our study defines the role of HOTAIR in PCSLCs expansion and the development of Doc resistance, providing rationale to build HOTAIR-targeted therapeutic strategies to overcome CRPC, especially Doc resistant CRPC.

## RESULTS

### HOTAIR induction in PCa cells leads to increased population of PCSLCs

**Figure 1:**
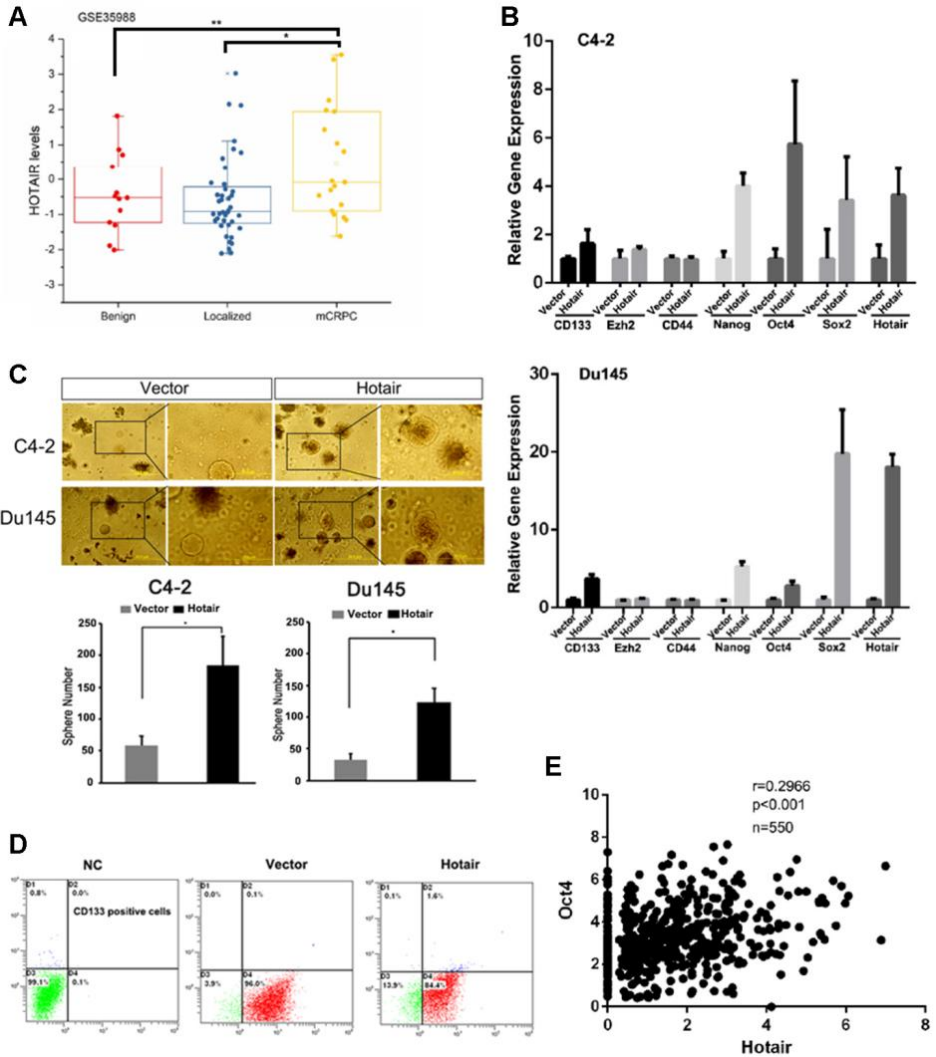
**HOTAIR induction in PCa cells leads to increased population of cancer stem cells.** (**A**) TCGA dataset showed that HOTAIR was highly expressed in metastatic CRPC samples. (**B**) HOTAIR overexpression in C4-2 cells (top) and Du145 cells (bottom) promoted the expression levels of several cancer stem cell markers. Gene expression was normalized to GAPDH mRNA. (**C**) HOTAIR overexpression in C4-2 cells and Du145 cells increased PCSLCs population. Top, Representative images of tumorspheres. Bottom, statistical analyses. (**D**) flow cytometry to analyze the population of CD133 positive cells before and after HOTAIR expression. (**E**) a positive correlation of HOTAIR and Oct4 was observed based on TCGA dataset. *p<0.05; **p<0.01.

To investigate the role of HOTAIR in prostate cancer development, we first analyzed its expression levels based on TCGA dataset (GSE35988). The analysis revealed that HOTAIR expression levels were significantly increased in metastatic CRPC (mCRPC) compared to those in localized PCa and benign tumors (Figure 1A). Owing to the fact that cancer stemness was tightly associated with cancer metastasis, we sought to examine whether HOTAIR was a causal factor determining cancer stemnness. To end this, we overexpressed HOTAIR in C4-2 cells and observed that induction of HOTAIR evidently upregulated the expression levels of several PCSLCs related markers such as CD133, Oct4, Sox2 and Nanog (Figure 1B). Sphere formation assay also confirmed that HOTAIR expressing C4-2 cells had better capacity to form tumorsphere (Figure 1C). Similar results were gained in Du145 cells showing that HOTAIR increased the population of PCSLCs (Figure 1B, 1C). Also, quantification analysis using flow cytometry showed that the population of C4-2 CD133 positive cells was shifted from 0.1% to 1.6% before and after HOTAIR overexpression (Figure 1D). Importantly, Oc4, a transcription factor that plays an essential role in pluripotency of embryonic stem cell or cancer stem cells, was positively corrected with HOTAIR according to the analysis from TCGA dataset (Figure 1E, r=0.2966, *p*<0.001). Together, all these data suggest that HOTAIR plays a critical role in maintaining the homeostatic stemnness of PCa.

### STAT3 activation is indispensable for the contribution of HOTAIR to cancer stemness

**Figure 2:**
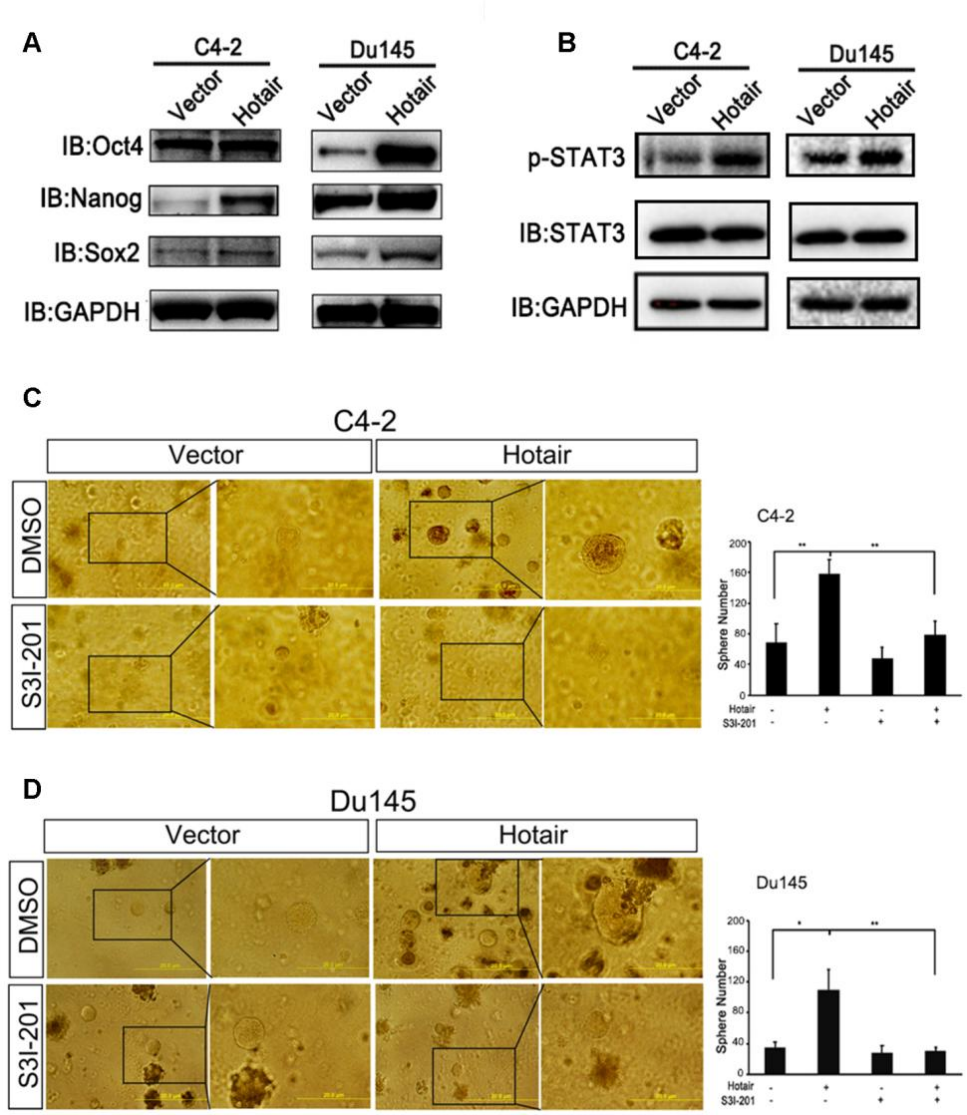
**STAT3 activation is indispensable for the contribution of HOTAIR to cancer stemness.** (**A**) Western blotting revealed that HOTAIR overexpression enhanced the expression levels of Sox2, Nanog and Oct4. GAPDH was used as internal control. (**B**) HOTAIR activated STAT3 signaling in both C4-2 cells and Du145 cells, monitored by p-STAT3 (Y705). GAPDH was internal control. (**C**, **D**) STAT3 inhibitor, S3I-201 (10 μM), could interrupt HOTAIR induced PCSLCs population in both C4-2 cells (**C**) and Du145 cells (**D**). Left, representative images of tumorspheres. Right, statistical analysis. *p<0.05; **p<0.01.

We first confirmed the expression levels of CSLCs markers induced by HOTAIR by western blotting. Data showed that HOTIAR could significantly enhance the expression levels of Sox2, Nanog and Oct4 in both C4-2 and Du145 cells (Figure 2A). Amounting evidence has demonstrated that STAT3 activation was necessary for PCSLCs development [13, 23, 24]. To test whether STAT3 was also involved in HOTAIR-induced PCSLCs population, we examined the phosphorylation levels of STAT3 at Y705 after the introduction of HOTAIR into C4-2 and Du145 cells. Figure 2B clearly showed that HOTAIR dramatically increased the phosphorylation levels of STAT3 at Y705, indicating STAT3 activation may participate in HOTAIR mediated PCSLCs development. Next, we wanted to test whether HOTAIR induced PCSLCs development could be interrupted by STAT3 inhibitor. Our result in C4-2 cells revealed that the HOTAIR-induced PCSLCs population could be blocked by the treatment of STAT3 inhibitor, S3I-201 (Figure 2C). Similar result was obtained from Du145 cells (Figure 2D). Collectively, these findings suggest that contribution of HOTAIR to prostatic cancer stemness is at least partly due to STAT3 activation.

### Mechanistic dissection of how HOTAIR activates STAT3 signaling

**Figure 3:**
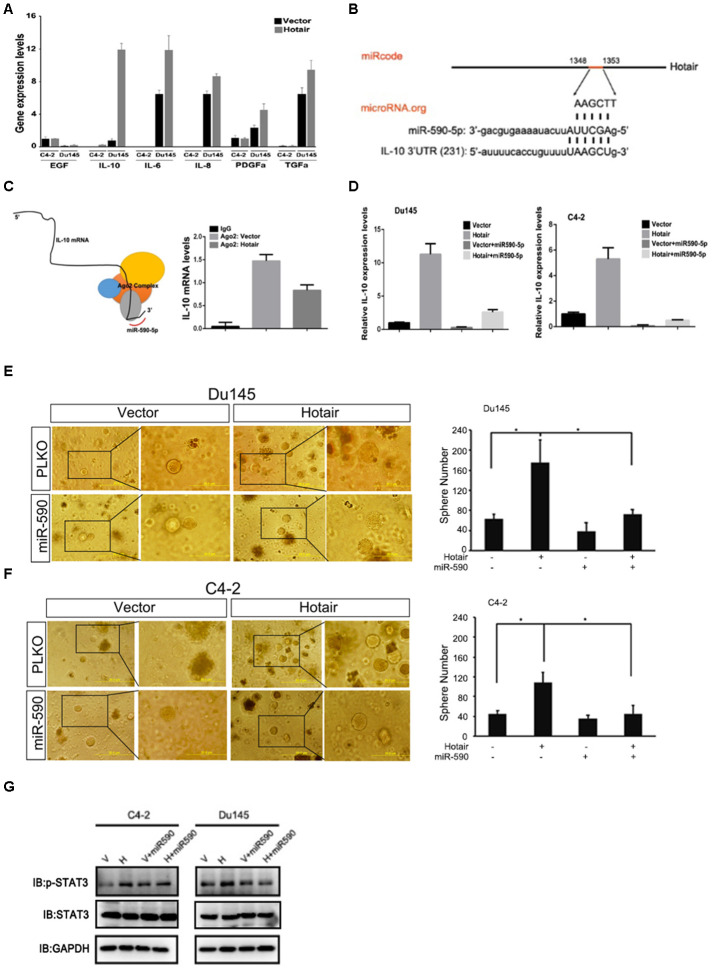
**Mechanism dissection of how HOTAIR activates STAT3 signaling.** (**A**) qPCR assay showed that IL-10 mRNA was consistently increased by HOTAIR in both C4-2 and Du145 cells. Gene expression was normalized to GAPDH mRNA. (**B**) Online software prediction displayed that HOTAIR and IL-10 were the potential targets of miR-590-5p. (**C**) top, interacting model of miR-590-5p, IL-10 mRNA and Ago2 complex. Bottom, Ago2 immunoprecipitation showed that less IL-10 mRNA was associated with Ago2 complex when HOTAIR was overexpressed. (**D**) miR-590-5p could impair HOTAIR induced IL-10 mRNA levels in C4-2 and Du145 cells. Gene expression was normalized to GAPDH mRNA. (**E**, **F**) miR-590-5p could rescue HOTAIR induced PCSLCs population in both C4-2 (**E**) and Du145 cells (**F**). Left, representative images of tumorspheres. Right, statistical analysis. (**G**) miR-590-5p could block HOTAIR induced STAT3 activity. GAPDH was loading control. *p<0.05; **p<0.01.

To find the underlying mechanism by which HOTAIR activated STAT3 signaling, we first examined expression levels of several upstream molecules involved in STAT3 signaling. We found that IL-10 but not other molecules was consistently upregulated when HOTAIR was overexpressed in C4-2 and Du145 cells (Figure 3A). HOTAIR can bind PRC2 complex to transcriptionally regulate gene expression. Interestingly, IL-10 induction by HOTAIR could not be rescued by adding Ezh2 inhibitor (data not shown), excluding the possibility that HOTAIR transcriptionally regulated IL-10. Long non-coding RNAs also can regulate gene expression via sponging its related miRNAs. By analyzing nucleotide sequence of HOTAIR and IL-10, we identified that HOTAIR and IL-10 are both potential targets of miR-590-5p (Figure 3B). In addition, Ago2 immunoprecipitation assay also showed that less IL-10 mRNA was associated with Ago2 complex when HOTAIR was overexpressed (Figure 3C), suggesting that HOTAIR may post-transcriptionally regulate IL-10 mRNA probably by acting as miRNA-590-5p sponge. To confirm this, we examined whether miR-590-5p could attenuate HOTAIR induced IL-10 expression. Results from C4-2 and Du145 cells exhibited that HOTAIR mediated IL-10 induction was blocked in the presence of miR-590-5p (Figure 3D). Consistently, HOTAIR-induced PCSLCs population was also impaired in the presence of miR-590-5p in both C4-2 and Du145 cells (Figure 3E–3F). Moreover, miR-590-5p bore the ability to deactivate STAT3 signaling induced by HOTAIR (Figure 3G). Collectively, all these data indicate that HOTAIR competes with IL-10 for miR-590-5p binding, leading to STAT3 activation.

### HOTAIR contributes to Docetaxel resistance of PCa cells

**Figure 4:**
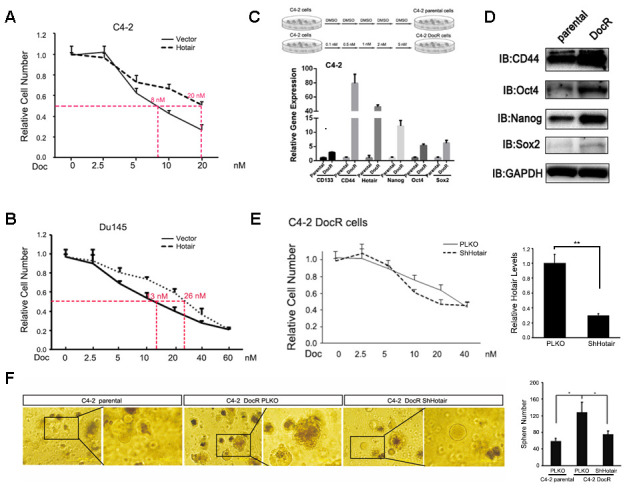
**HOTAIR contributes to Docetaxel resistance of PCa cells.** (**A**, **B**) overexpression of HOTAIR caused Doc resistance in C4-2 (**A**) and Du145 cells (**B**). (**C**) Top, carton showed how C4-2 DocR cells were established. Bottom, C4-2 DocR cells expressed higher levels of HOTAIR and cancer stem cells markers. Gene expression was normalized to GAPDH mRNA. (**D**) Cancer stem cells markers (Sox2, Nanog and Oct4) were overexpressed in C4-2 DocR cells compared to parental cells. GAPDH was internal control. (**E**) left, knockdown of HOTAIR restore Doc sensitivity of C4-2 DocR cells; right, knockdown efficiency of HOTAIR in C4-2 DocR cells. (**F**) knockdown of HOTAIR impaired the tumor sphere forming ability of C4-2 DocR cells. Left, representative images of tumorspheres. Right, statistical analysis. *p*<0.05; **p<0.01.

Given the drug resistant property of PCSLCs, we sought to test whether HOTAIR contributed to Doc sensitivity in PCa cells. Frist, we found that overexpression of HOTAIR in C4-2 and Du145 cells led their cells to be more resistant to Doc treatment (Figure 4A, 4B). To further confirm the role of HOTAIR in the development of DocR resistance, we established C4-2 DocR cells by continuously adding various concentrations of Doc for more than 6 months. Of note, C4-2 DocR cells were more senescent like compared to C4-2 parental cells. Q-PCR result showed that HOTAIR and PSCLCs markers were dramatically induced in C4-2 DocR cells compared to the corresponding C4-2 parental cells (Figure 4C, 4D). Then we knocked down HOTAIR by shRNAs (Figure 4E, right), and found that deficiency of HOTAIR in C4-2 DocR cells could restore Doc sensitivity (Figure 4E, left), suggesting HOTAIR was indeed one of causal factors determining the development of Doc resistance. Importantly, C4-2 DocR cells had better capacity to form tumorsphere compared to C4-2 parental cells, which could be impaired by the knockdown of HOTAIR (Figure 4F). Together, all these data imply that the contribution of HOTAIR to Doc resistance was attributable to its ability to increase the population of cancer stem cells.

### HOTAIR promotes tumor growth of xenografted PCa mouse model

**Figure 5:**
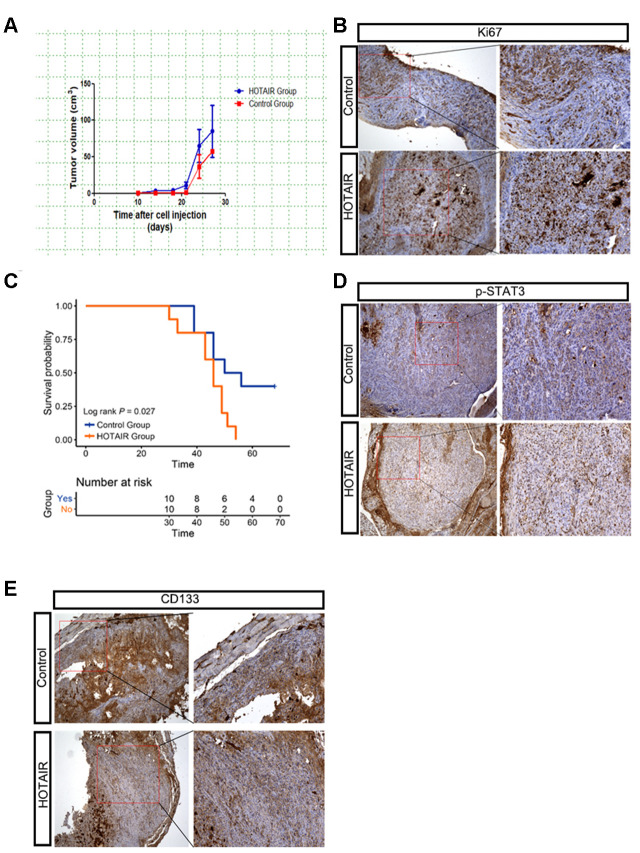
**HOTAIR promotes tumor growth of xenografted PCa mouse model.** (**A**) HOTAIR Du145 tumors grew faster than control ones. Tumor volume=1/2*short axis^2^*long axis. (**B**) IHC staining of Ki67 indicated that HOTAIR Du145 tumors had higher proliferating rate. (**C**) HOTAIR Du145 mice had poor survival rate compared to control cohorts. (**D**) IHC staining of p-STAT3. (**E**) IHC staining of CD133.

To verify the role of HOTAIR in the *in vivo* mouse model, we subcutaneously implanted Du145-vector cells (1 x10^6^) and Du145-HOTAIR cells (1 x10^6^) into 6-week male nude mice. After 10 days, tumors were monitored and measured by caliper every three days. Data showed that HOTAIR Du145 tumors grew relatively faster than the control ones (Figure 5A). Consistently, the positive staining of proliferation marker Ki67 was significantly increased in Du145 HOTAIR tumors compared to that in controls (Figure 5B). We also noticed that Du145 HOTAIR mice had poor survival rate compared to control cohorts (Figure 5C, p=0.027), suggesting Du145 HOTAIR tumors were more malignant than control ones. To link these *in vivo* results with our *in vitro* findings, we examined STAT3 signaling and PCSLCs population in the xenograted tumors, monitored by p-STAT3 (Y705) and CD133 respectively. Data revealed that the phosphorylation levels of STAT3 at Y705 were clearly increased in Du145 HOTAIR tumors (Figure 5D). Meanwhile, CD133 positive PCSLCs were also increased in Du145 HOTAIR tissues when compared to Du145 vector tissues (Figure 5E). In summary, these findings indicate that HOTAIR promotes xenografted PCa tumor growth by activating STAT3 signaling and increasing PCSLCs population.

## DISCUSSION

PCa remains the major leading cause of cancer related death among men worldwide [1]. Although AR plays central role in PCa development, PCSLCs are AR negative cells and they are inherently resistant to any AR targeted therapies. Owing to the fact that there are very limited strategies for the depletion of PCSLCs, scientists are urgently identifying novel targets in order to develop better treatments towards PCSLCs. In this study, we found that HOTAIR overexpression could expand the PCSLCs population, leading to Doc resistance. Mechanistically, HOTAIR activated STAT3 signaling via upregulating IL-10 mRNA levels. HOTAIR sponged miR-590-5p and prevented it from the binding with the 3’UTR of IL-10 mRNA. Importantly, *in vivo* animal study also confirmed that HOTAIR had better capacity to promote tumor growth. Overall, our data define the role of HOTAIR in PCSLCs development and provide rationale to develop HOTAIR based therapy to overcome PCa progression. Doc was approved to treat metastatic CRPC patients since 2014. However, Doc resistance will eventually occur and become a clinical problem. Therefore, understanding the underlying mechanisms responsible for Doc resistance is of necessity to improve its efficacy. For instance, KDM5D was one of factors involving in Doc resistance development [25]. KDM5D negatively regulated AR expression levels via reducing the H3K4me3 levels at its promoter locus. Knockdown of KDM5D led to AR activation and caused docetaxel insensitivity [25]. AR variant, AR-v7, has also been reported as a causal factor in the development of Doc resistance [26]. Here, our data also showed that HOTAIR depletion could restore Doc sensitivity. However, how to complete the goal of specifically targeting lncRNAs is still a scientific problem and remains a scientific interest in the future.

Amounting evidence suggests that HOTAIR plays a tumor promoting role in CRPC stage [20]. HOTAIR interacted with AR protein and prevented it from MDM2 mediated protein degradation [20]. Consequently, HOTAIR overexpression could stabilize AR protein to provide survival signal for PCa cells in the castrated condition. Here, we supplemented that HOTAIR overexpression could also activate STAT3 signaling to expand PCSLCs population, which may be explained by the heterogeneous functions of HOTAIR in PCa development. The classical role of HOTAIR depended its interaction with PRC2 complex to transcriptionally regulate gene expression. However, we noticed that the contribution of HOTAIR to PCSLCs development was not dependent of PRC2 complex because Ezh2 (one important PRC2 component) inhibitor failed to block HOTAIR mediated IL-10 upregulation and PCSLCs expansion. We also found that HOTAIR could not interact with STAT3 (data not shown). Interestingly, HOTAIR performed unusual way to increase STAT3 activity by working as miR-590-5p sponge and promoting IL-10 expression. Actually, the sponging ability of HOTAIR has been recognized recently. HOTAIR could sponge miR-138, miR-200c, miR-204 and miR-217 to increase several oncogenes to promote renal cell carcinoma progression [27], implying that HOTAIR may have different sponging potentials in different tissues.

Our results clearly demonstrated that HOTAIR was over-induced in C4-2 Doc resistant cells. Previous study pointed that HOTAIR was one of AR-suppressed lncRNAs [20]. Anti-androgen could release HOTAIR from the repression of AR. Another article also suggested that Doc treatment could decrease AR expression. In this case, chronic Doc treatment led to AR reduction and expanded the population of PCSLCs so that higher levels HOTAIR were observed in Doc resistant cells.

In summary, we found HOTAIR could activate STAT3 signaling to increase PCa malignancy *in vitro* and *in vivo* and targeting HOTAIR may deplete PCSLCs population and increase Doc efficacy.

## MATERIALS AND METHODS

### Cell culture

Phoenix 293T, 293T, PCa C4-2 and Du145 cells were purchased from Cell Bank of Chinese Academy Of Science (Shanghai, China). C4-2 and Du145 cells were maintained in RPMI-1640 Medium supplemented with 10% FBS (Gibco), penicillin (100 units/ml), streptomycin (100 μg/ml) and 1% L-glutamine. And phoenix 293T and 293T cells were cultured in DMEM with 10% FBS (Gibco), penicillin (100 units/ml), streptomycin (100 μg/ml) and 1% L-glutamine. All cells were kept in a 5% CO2 humidified incubator at 37°C.

### Xenograft mouse model

Du145-vector or Du145-HOTAIR cells (1X10^6^) were subcutaneously injected into 6-weeks male nude mice with equal amount of matrigel. 10 days later, tumors were monitored and measured by caliper every three days. Tumor tissues were stored in liquid nitrogen for further use.

### *In vitro* DocR cell line establishment

C4-2 cells were continuously exposing to various concentrations of Doc (0.1, 0.5, 1, 2, 5 nM) for more than 6 months. Simultaneously, C4-2 cells exposed to DMSO were considered as C4-2 parental cells. The property of Doc resistance was determined by the responses of C4-2 parental and C4-2 DocR cells to Doc treatment. Then C4-2 DocR cells were cultured with 10% FBS RIPM supplemented with 5 nM Doc.

### Retroviral generation

LZRS-HOTAIR and control vector (20 μg) were transfected into Phoenix 293 T to generate lentivirus. 48 hours later, viruses were collected and infected PCa cells in the presence of 8 μg/ml polybrene.

### Lentivirus generation

PLKO-shHOTAIR, PLKO-miR-590-5p and PLKO-vector were transfected into 293T cells with packaging plasmids: PAX2 and D2G. 48 hours later, viruses were collected and infected PCa cells in the presence of 8 μg/ml polybrene. Infected cells were selected with 1 μg/ml puromycin for one week before experiments.

### Western blotting

PCa cells were lysed in cold RIPA buffer. Equal amount of protein was loaded and separated on 10% SDS/PAGE gel. Protein samples were transferred onto PVDF membranes (Millipore). After being blocked in 5% milk for 1-2 hour, the membranes were probed with specific primary antibodies overnight at 4°C: p-STAT3 (Y705) (9131, Cell signaling), GAPDH (SC-32233, Santa Cruz) and followed by incubation with HRP-conjugated secondary antibody for 1 hour at room temperature. After extensive wash with TBST, blots were visualized using ECL system (Thermo Fisher Scientific, Waltham, MA).

### RNA isolation and Real time PCR

Cells were lysed by trizol reagent (Invitrogen) and RNAs were extracted. 1 μg RNA was used to make cDNA by using Superscript III reverse transcription system (Invitrogen). Diluted cDNA (20 times) was used to perform quantitative real-time PCR (qRT-PCR) using a Bio-Rad CFX96 system with SYBR green. The mRNA levels of GAPDH were use as internal control. Primers were listed in Supplementary Table 1.

### Ago2 RNA Immunoprecipitation (RIP)

C4-2 cells with HOTAIR manipulation were lysed in ice-cold lysis buffer supplemented with RNase inhibitor. After centrifugation, 10 mg of the supernatant was cleared by protein A/G beads for 1 hour and incubated with Ago2 antibody overnight at 4 ºC. Then the beads pre-blocked by 15 mg/ml BSA were added to the antibody-lysate mixture and incubated for another 2 hours. The RNA/antibody complex was washed with RIPA buffer supplemented with RNase inhibitor, protease inhibitor cocktail, for four times. The RNA was extracted using Trizol (Invitrogen) according to the manufacturer’s protocol and subjected to qRT-PCR analysis.

### Sphere formation assay

After gene manipulation, 5 x 10^3^ C4-2 or Du145 cells were suspended in serum free RPMI medium and equally mixed with Matrigel (Corning). 100 μL mixture was loaded to 24-well plate and supplemented with 1 mL medium. Two weeks later, spheres were captured by microscopic machine.

### Immunohistochemical (IHC) staining

Antigen retrieval was performed on rehydrated tissue sections in boiling citrate buffer (pH 6.0) for 30 min. After being treated with 3% H_2_O_2_ and blocking buffer (5% normal goat serum in PBS), tissue sections were incubated with p-STAT3 (Y705) (9131, Cell signaling), Ki67 (BD pharmingen) or CD133 (Proteintech) at 4 °C overnight. Then biotin-labeled secondary antibody was added for another 30 min, followed by 30 min incubation of streptavidin-horseradish peroxidase (HRP) (PK-4000, Vectastain, Burlingame, CA) and signals were visualized by peroxidase substrate DAB kit (SK-4100, Vectastain, Burlingame, CA).

### Statistics

Significance of correlations was done using GraphPad Prism software. Data were presented as mean ± SE. Differences were analyzed with the Student t test and significance was set at P <0.05.

## Supplementary Material

Supplementary Table 1
