# Supplementary material for: HOTAIR expands the population of prostatic cancer stem-like cells and causes Docetaxel resistance via activating STAT3 signaling
Source: Aging (Albany NY). 2020 Jul 13;12(13):12771–82. doi: 10.18632/aging.103188 (PMC7377851; doi:10.18632/aging.103188)
Supplement: Supplementary Table 1 [file aging-12-103188-s001.pdf]

SUPPLEMENTARY TABLE

Supplementary Table 1. Primers used in real time PCR.

Gene Name	Sequence (5'-3')
HOTAIR	F: GGCGGATGCAAGTTAATAAAAC
	R: TACGCCTGAGTGTTCACGA
SOX2	F: CTCGTGCAGTTCTACTCGTCG
	R: AGCTCTCGGTCAGGTCCTTT
NANOG	F: TCCCGAGAAAAGATTAGTCAGCA
	R: AGTGGGGCACCTGTTTAACTT
OCT4	F: GCATTCAAACTGAGGCACCA
	R: AGCTTCTTTCCCCATCCCA
CD133	F: CACTTGATGCCACTGCCAAA
	R: TGACACTGAACGTAATGCCCAT
EZH2	F: AATCAGAGTACATGCGACTGAGA
	R: GCTGTATCCTTCGCTGTTTCC
GAPDH	F: GGAGCGAGATCCCTCCAAAAT
	R: GGCTGTTGTCATACTTCTCATGG
